# Exploratory small RNA sequencing of human placental miRNAs identifies candidates associated with congenital transmission of Trypanosoma cruzi

**DOI:** 10.21203/rs.3.rs-10217677/v1

**Published:** 2026-07-14

**Authors:** Andrea Diestra, Alejandra Pando-Caciano, Carla A. Apaza-Quiroz, Vanessa Adaui, Viviana Pinedo-Cancino, Sylvia S. Sanchez, Monica Mugnier, Robert H. Gilman, Natalie M. Bowman, Monica J. Pajuelo

**Affiliations:** Universidad Peruana Cayetano Heredia; Universidad Peruana Cayetano Heredia; Universidad Peruana Cayetano Heredia; Universidad Peruana de Ciencias Aplicadas (UPC); Universidad Nacional de la Amazonía Peruana; Johns Hopkins University Bloomberg School of Public Health; Johns Hopkins Bloomberg School of Public Health; Johns Hopkins University Bloomberg School of Public Health; University of North Carolina at Chapel Hill; Universidad Peruana Cayetano Heredia

**Keywords:** Chagas Disease, Infectious Disease Vertical Transmission, MicroRNAs, Placenta, Trypanosoma cruzi

## Abstract

**Background:**

Congenital transmission remains a major source of new Chagas disease cases, yet the underlying molecular mechanisms remain poorly understood. This exploratory study aimed to identify human placental microRNAs (miRNAs) associated with congenital transmission of *Trypanosoma cruzi*.

**Methods:**

Placental samples from *T. cruzi*-infected mothers were classified according to congenital transmission status. Small RNA sequencing was performed on 31 placental samples, including 13 from transmitting mothers and 18 from non-transmitting mothers. Differential miRNA expression was assessed using DESeq2 with Benjamini-Hochberg false discovery rate correction. Candidate miRNAs were selected based on magnitude and significance of differential expression, or concordance with prior evidence from placental *T. cruzi* infection models. Selected candidates were subsequently analyzed by RT-qPCR in 15 placental samples, including 5 from transmitting mothers and 10 from non-transmitting mothers.

**Results:**

Small RNA sequencing analysis identified hsa-miR-155–5p as the only significantly upregulated miRNA in placentas from transmitting mothers compared with placentas from non-transmitting mothers (log_2_ fold change = 1.26; adjusted p-value = 0.024); however, RT-qPCR assessment failed to confirm it at a significant level (log_2_ fold change = 0.57, 1.48-fold difference; p-value = 0.426). Other miRNAs, hsa-miR-193a-5p and hsa-miR-187–3p, showed higher relative expression in transmitting mothers, with estimated log_2_ fold changes of 2.31 and 1.53, respectively (p = 0.032 and p = 0.043). hsa-miR-526b-3p showed a modest decrease in relative expression in transmitting mothers (log_2_ fold change = −0.46; p = 0.043).

**Conclusion:**

Placental miRNA expression profiling of naturally infected mothers identified miRNA candidates potentially relevant to congenital transmission of *T. cruzi*. Given the capacity of miRNAs to regulate broad gene expression networks that influence placental biology, these results underscore the need for transcriptomic and functional studies to elucidate their regulatory relevance during *T*. infection and evaluate their potential as biomarkers for congenital transmission risk.

## BACKGROUND

Chagas disease, caused by the protozoan parasite *Trypanosoma cruzi*, remains a major neglected tropical disease in Latin America, where an estimated 6–7 million people are infected and millions more remain at risk (1). Although vector-borne transmission predominates in endemic areas, congenital transmission has emerged as an increasingly relevant route of infection, particularly in the context of migration among chronically infected women of reproductive age (2). Across the Americas, congenital transmission occurs in 1–10% of pregnancies among infected women (3, 4) representing a sustained source of new cases independent of vector exposure.

The clinical presentation of congenitally infected neonates is highly heterogeneous. Approximately 60 to 90% are asymptomatic at birth, while others may present with low birth weight, low Apgar scores, hepatosplenomegaly, anemia, or hepatic dysfunction (5, 6). Importantly, untreated infants, including those who are asymptomatic at birth, remain at risk of developing chronic cardiac or gastrointestinal disease later in life (7). Early antiparasitic treatment with benznidazole or nifurtimox during the first year of life is highly effective, with cure rates approaching 100% (8, 9).

Although only a small proportion of infected mothers transmit the *T. cruzi* parasite to their newborns (3, 10), early diagnosis of congenital infection remains challenging. Current screening strategies rely on sequential parasitological, serological, and molecular testing over several months, and routine neonatal screening is not consistently implemented in many settings (6). These diagnostic limitations highlight the need for complementary approaches to support the early identification of congenital infection.

The placenta serves as a key biological interface between maternal infection and fetal exposure. Congenital transmission of *T. cruzi* requires parasite invasion of placental tissues, modulation of local immune responses, and disruption of trophoblast barrier integrity (11). In this context, characterizing the molecular signatures associated with congenital transmission of *T. cruzi* may provide insights into the cellular pathways involved in congenital Chagas disease. These insights may ultimately help identify candidate biomarkers for future evaluation. MicroRNAs (miRNAs), small non-coding RNAs that regulate gene expression post-transcriptionally (12), are key modulators of placental development, immune signaling, inflammatory pathways, and host-pathogen interactions (13). Altered placental miRNA expression has been implicated in pregnancy complications (14) and infectious diseases (15), suggesting that these regulatory molecules may influence susceptibility or resistance to *T. cruzi* transplacental passage.

Despite the epidemiological importance of congenital Chagas disease, placental miRNA expression profiles associated with congenital transmission of *T. cruzi* remain poorly characterized. Altered miRNA expression has been previously reported in human placental explants experimentally infected with *T. cruzi* (16, 17), suggesting the potential relevance of miRNAs as candidate biomarkers for placental infection and congenital transmission risk. However, placental miRNA expression profiles from naturally infected mothers stratified by transmission status have not yet been described. Therefore, this study aimed to identify differentially expressed placental miRNAs associated with natural congenital transmission of *T. cruzi* using small RNA sequencing and to assess the relative abundance of selected candidate miRNAs by reverse transcription quantitative polymerase chain reaction (RT-qPCR).

## METHODS

### Study Design and Sample Source

This study used placental tissue samples and associated clinical and diagnostic data from two cohorts of *T. cruzi*-infected women who delivered at Hospital De La Mujer Dr. Percy Boland, a reference maternity hospital in Santa Cruz de la Sierra, Bolivia, during two study periods: 2022–2023 and 2024–2025. Participants in the parent cohorts were recruited as part of studies aimed at improving diagnostic approaches for congenital Chagas disease (10, 18).

Within the parent cohort studies, maternal *T. cruzi* infection was initially screened using rapid diagnostic tests (Chagas Detect^™^ Plus Rapid Test, InBios International, Inc.) and subsequently confirmed by serological testing using enzyme-linked immunosorbent assay (ELISA) (Chagatest ELISA Recombinante v.3.0, Wienner Lab.) (19). Congenital infection status was determined at birth by qPCR performed on peripheral blood or umbilical cord blood samples collected from the newborns (20, 21).

As part of the parent cohort protocols, placental tissue samples were collected at delivery and stored at −20°C in DNA/RNA Shield^™^ (Zymo Research, Irvine, CA, USA) or RNA*later*^™^ (ThermoFisher Scientific, Waltham, MA, USA) for subsequent laboratory analyses. Samples were classified according to recorded neonatal qPCR results into two groups: placentas from transmitting mothers, whose newborns had positive qPCR results for *T. cruzi*, and placentas from non-transmitting mothers, whose newborns tested negative by qPCR.

All available placenta samples from transmitting mothers were selected and matched to samples from non-transmitting mothers based on maternal age range (+/− 2 years) and mode of delivery. Based on the availability of eligible placental specimens from infants with confirmed congenital transmission status, a total of 31 samples were included in the small RNA sequencing analysis: 13 samples from transmitting mothers (9 from the first cohort and 4 from the second cohort) and 18 samples from non-transmitting mothers (9 from each cohort). Sequencing for the first and second cohorts was performed independently in two batches due to sample availability.

RT-qPCR assessment was performed in 15 placental samples from participants enrolled in the second cohort (5 from transmitting mothers; 10 from non-transmitting mothers), since no RNA was available from the first cohort samples used for sequencing. This set included the 13 second-cohort samples used for small RNA sequencing (4 from transmitting mothers and 9 from non-transmitting mothers), alongside two additional samples not included in the sequencing analysis (1 from a transmitting mother and 1 from a non-transmitting mother). These two additional samples were included after sequencing.

### RNA Extraction and Quality Assessment

Total RNA was extracted from placental tissue using the mirVana^™^ miRNA Isolation Kit (Invitrogen, Waltham, MA, USA), following the manufacturer’s protocol. RNA concentration was quantified using a Qubit^™^ 4 Fluorometer (Thermo Fisher Scientific, Waltham, MA, USA). RNA integrity was assessed using an Agilent 2100 TapeStation system (Agilent Technologies, Santa Clara, CA, USA). RNA samples with an RNA integrity number equivalent (RINe) ≥ 7.0 were included in the small RNA sequencing analysis.

### Small RNA Library Preparation and Sequencing

Small RNA libraries were prepared using the QIAseq miRNA Library Kit (QIAGEN, Hilden, Germany), according to the manufacturer’s instructions. Libraries were pooled and sequenced on an Illumina NovaSeq 6000 platform (Illumina, Inc., San Diego, CA, USA) using a 2 × 150 bp paired-end configuration, targeting approximately 20 million reads per sample.

Small RNA library preparation and sequencing were performed by Admera Health (South Plainfield, NJ, USA) in two experimental batches. Batch allocation was recorded for each sample and considered in subsequent exploratory and differential expression analyses.

Raw sequencing reads were processed using the Galaxy Europe platform v24.1 (22). Read quality was evaluated using FastQC v0.12.1 (23) and summarized using MultiQC v1.21 (24). Adapter sequences were removed and reads shorter than 19 nucleotides were discarded before downstream processing with miRDeep2. Processed reads were mapped using miRDeep2 Mapper v2.0.1, and known human miRNAs were quantified using miRDeep2 Quantifier v2.0.1 (25) with human miRNA reference sequences from miRBase v22.1. The resulting raw count matrix was used for downstream differential expression analysis.

### Exploratory Analysis of Global miRNA Expression Patterns and Batch Effects

Exploratory analyses of the small RNA sequencing data were performed in R version 4.3.3 in the RStudio integrated development environment (2023.12.1 + 402) (Posit Software, PBC, Boston, MA, USA). Principal component analysis (PCA) was conducted on variance-stabilized expression data to examine global miRNA expression patterns and identify potential sources of technical and biological variation. Variance-stabilized expression values were generated from raw miRNA counts using the *varianceStabilizingTransformation* function in DESeq2 package (v1.42.1), which transforms count data to reduce the dependence of variance on mean expression levels. Principal components were calculated using the *prcomp* function and visualized using ggplot2 (v3.5.2). Associations between principal components and continuous cohort characteristics were evaluated using Pearson’s correlation coefficients, whereas associations with categorical characteristics were evaluated by analysis of variance (ANOVA), with η^2^ used to quantify the proportion of variance explained by each variable. To account for batch-related technical variation in the exploratory analysis, batch-corrected normalized expression values were obtained using the limma package (v3.58.1) *removeBatchEffect()* function, and PCA-based assessments were repeated following batch correction.

### Differential expression analysis

Differential miRNA expression analysis was performed using the DESeq2 package. Prior to differential expression analysis, miRNAs with a mean normalized expression level (baseMean) of at least 5 normalized counts in both the transmitting and non-transmitting groups were retained. This filtering step was applied to remove features with insufficient count information, which are more susceptible to technical variability and provide limited statistical power for reliable differential expression testing. Raw count data were normalized using the median-of-ratios method implemented in DESeq2 to account for differences in sequencing depth and library composition across samples.

Differential expression between placental samples from transmitting and non-transmitting mothers was assessed using a negative binomial generalized linear model. Sequencing batch was included as a covariate in the DESeq2 model to account for batch-related technical variation. Statistical significance was evaluated using the Wald test, and p-values were adjusted for multiple comparisons using the Benjamini–Hochberg false discovery rate (FDR) procedure. miRNAs with an absolute log_2_ fold change > 1 (i.e. at least a 2-fold expression difference) and an adjusted p-value < 0.05 were considered differentially expressed. Differential expression results were visualized using volcano plots and heatmaps generated with ggplot2 (v3.5.2) and pheatmap (v1.0.12), respectively.

### Selection of candidate miRNAs for RT-qPCR assessment

Ten candidate miRNAs for RT-qPCR assessment were selected from the small RNA sequencing results using a sequential prioritization strategy, due to resource constraints. Candidate selection integrated the results of differential expression analyses performed separately for each sequencing batch and for the combined dataset. Selection criteria were applied hierarchically. Candidates were required to meet a log_2_ fold change ≥ 1.0 for upregulated miRNAs or ≤ − 1.0 for downregulated miRNAs. Initial priority was given to miRNAs that met the statistical significance threshold (adjusted p-value < 0.05). To ensure that potentially relevant candidate miRNAs were not overlooked, additional miRNAs with a nominal p-value < 0.05 were also considered. Furthermore, previous literature evidence linking specific miRNAs to *T. cruzi* infection in placental explants was considered as an additional criterion for candidate selection.

### Selection of reference miRNAs for RT-qPCR normalization

Two candidate reference miRNAs for RT-qPCR data normalization were preselected from the small RNA sequencing data based on minimal expression differences between samples from transmitting and non-transmitting mothers, defined as a p-value > 0.05 and a log_2_ fold change between − 0.1 and 0.1. RT-qPCR was then performed for the 12 selected miRNAs, including the candidate miRNAs identified from the differential expression analyses and the two candidate reference miRNAs.

Expression stability was assessed using the geNorm algorithm (26), as implemented in the Clarida Reference Gene Finder (v2026.06.0)(27). geNorm calculates the gene expression stability value (M) through pairwise comparison of each candidate miRNA with all other evaluated candidates, with lower M values indicating greater expression stability. M-value interpretation was based on the thresholds proposed by Hellemans et al. (28), where M values < 0.5 are typically observed for stable reference genes in relatively homogeneous sample panels.

To improve normalization robustness, a multi-reference normalization strategy was applied, consistent with the geNorm approach recommending the use of multiple stable reference genes for normalization whenever feasible (26). Accordingly, the final normalization factor was calculated using the selected reference miRNAs.

### RT-qPCR assessment of selected miRNAs

Reverse transcription was performed using the TaqMan^™^ MicroRNA Reverse Transcription Kit (Applied Biosystems, Waltham, MA, USA), according to the manufacturer’s instructions, using 1 μg of total RNA per reaction. The resulting cDNA was diluted 1:10 and amplified using the StepOnePlus^™^ Real-Time PCR System (Applied Biosystems, Waltham, MA, USA). The expression levels of the selected candidate miRNAs were assessed using individual TaqMan^™^ MicroRNA Assays (Applied Biosystems, Waltham, MA, USA), according to the manufacturer’s protocol. qPCR reactions were performed in duplicate for each sample under the following thermal cycling conditions: 20 s at 95°C followed by 40 cycles of 1 s at 95°C and 20 s at 60°C. The list of miRNAs evaluated and their corresponding TaqMan assay IDs are provided in Table S1.

Relative expression fold changes were estimated using the comparative Cq method described by Livak and Schmittgen (2001) (29). For each sample, the Cq value of each target miRNA was normalized to the mean Cq value of the selected endogenous reference miRNAs (ΔCq = Cq_target miRNA - mean Cq_reference miRNAs). For each target miRNA, ΔΔCq was estimated using the Hodges-Lehmann estimator of the between-group shift in ΔCq values, defined as the median of all pairwise differences between transmitting and non-transmitting samples. Relative fold change was then calculated as 2^−ΔΔCq^, with the log_2_ fold change equal to −ΔΔCq. In line with recommendations emphasizing the importance of reporting uncertainty in qPCR-derived fold change estimates (30), fold changes were reported alongside their corresponding 95% confidence intervals. For graphical representation, ΔCq values were transformed to −ΔCq and plotted by group using boxplots.

### Statistical data analysis

Available maternal and neonatal characteristics recorded in the parent cohort databases were summarized according to congenital transmission status. Maternal age, number of pregnancies, and number of live births were reported as medians and interquartile ranges (IQRs) and compared between transmitting and non-transmitting mothers using the Wilcoxon rank-sum test. Mode of delivery, newborn sex, and neonatal qPCR results for *T. cruzi* in peripheral blood and cord blood samples were reported as absolute frequencies and percentages. Mode of delivery was compared between groups using Fisher’s exact test, whereas newborn sex was compared using the chi-square test.

For the RT-qPCR assessment, ΔCq values of the selected candidate miRNAs were compared between placental samples from transmitting and non-transmitting mothers using the Mann-Whitney U test for independent samples. Significance level was set at 0.05. All RT-qPCR statistical analyses were performed in R using functions from the dplyr and stats packages. Graphical outputs were generated using ggplot2.

All statistical analyses were performed using R version 4.3.23 in the RStudio integrated development environment (version 2024.12.0 + 467) (Posit Software, PBC, Boston, MA, USA).

## RESULTS

### Study population characteristics

A total of 31 placenta samples from *T. cruzi*-infected pregnant women were included in the study, comprising 13 transmitting and 18 non-transmitting mothers. Maternal and neonatal characteristics were comparable between groups (Table 1). Male newborns accounted for 77% of births in the transmitting group and 50% in the non-transmitting group; however, this difference was not statistically significant (p = 0.130).

Among newborns in the transmitting group, *T. cruzi* DNA was detected by qPCR in peripheral blood or cord blood samples, with Cq values ranging from 15.63 to 29.18 and from 12.50 to 27.02, respectively. No amplification was observed in newborns from the non-transmitting group (Table 1).

### Global Placental miRNA Expression Patterns and Assessment of Batch-Related Variation

Small RNA sequencing yielded high-quality data, with an average of 75–80% of bases achieving a Phred quality score above 30 (Q30), supporting reliable downstream differential expression analysis.

A total of 442 miRNAs were detected across all placental samples prior to differential expression analysis. Among them, 418 miRNAs were detected in both transmitting and non-transmitting groups, while 14 were detected exclusively in the transmitting group and 10 only in the non-transmitting group.

**Figure 1:**
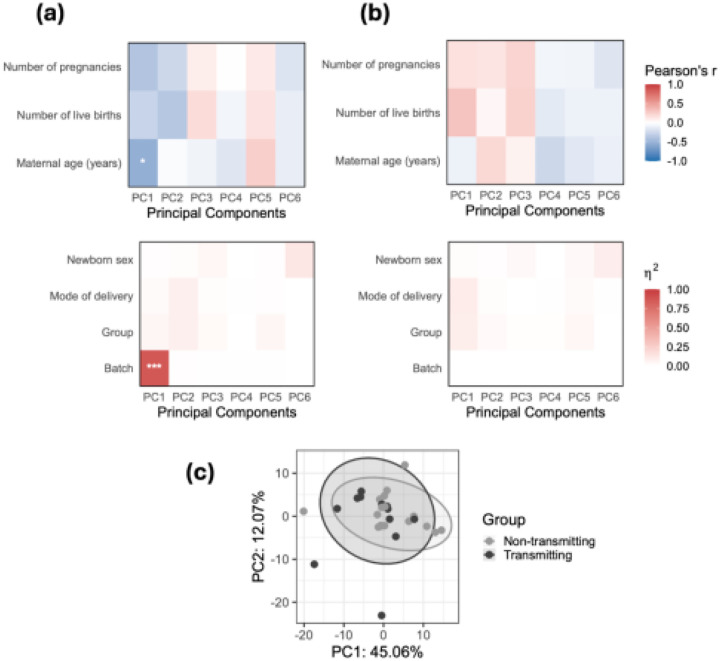
Global placental miRNA expression patterns and their association with cohort characteristics before and after batch correction.

PCA was performed to examine global placental miRNA expression patterns and assess the association of technical and clinical variables with overall expression variability. Before batch correction, PC1 accounted for 86.18% of the total variance and was strongly associated with sequencing batch, indicating a substantial influence of batch-related technical variation on the global miRNA expression profile (Fig. 1a). After batch correction, the association between sequencing batch and the principal components was substantially reduced, with batch explaining less than 1% of the variance in both PC1 and PC2, consistent with effective mitigation of batch-related variation. Following batch correction, none of the evaluated clinical variables, including number of pregnancies, live births, maternal age, newborn sex, or mode of delivery, showed a significant association with the principal components (Fig. 1b). Congenital transmission status was not strongly associated with any principal component, suggesting that transmission status was not a major driver of the global placental miRNA expression profile (Fig. 1c).

Heatmaps show the associations between the first six principal components (PC1-PC6) and cohort characteristics before (a) and after (b) batch correction. Continuous variables are represented by Pearson’s correlation coefficients (r), whereas categorical variables are represented by η^2^ values derived from ANOVA. Asterisks indicate statistically significant associations after multiple-testing correction. (c) Principal component analysis of batch-corrected miRNA expression data, with samples colored by group; group refers to congenital transmission status.

### Differential expression analysis and selection of candidate miRNAs for RT-qPCR assessment

Although congenital transmission status did not explain a large proportion of the global variance in placental miRNA expression profiles, differences in the expression of individual miRNAs between samples from transmitting and non-transmitting mothers were further assessed using DESeq2, with sequencing batch included as a covariate in the model. This analysis revealed one miRNA downregulated in transmitting samples (hsa-miR-498–3p: log_2_ fold change = −1.06; p-value = 0.003) and seven miRNAs upregulated in placentas from transmitting mothers with log_2_ fold change ≥ 1 and nominal p-values ≤ 0.05 (Table S2). Among these, hsa-miR-155–5p (log_2_ fold change = 1.26; adjusted p-value = 0.024) was the only miRNA significantly upregulated in placentas from transmitting mothers following multiple-testing correction.

**Figure 2:**
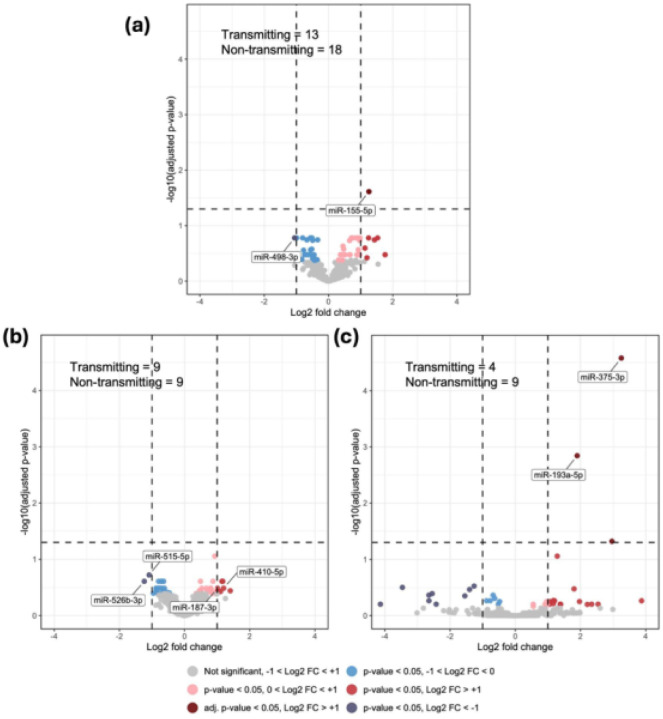
Volcano plots of placental miRNA differential expression between transmitting and non-transmitting mothers based on small RNA sequencing. Each point represents one detected miRNA, plotted according to log_2_ fold change and −log10 adjusted p-value. Labeled miRNAs correspond to candidates selected from each analysis. (a) Combined analysis including both sequencing batches. (b) First cohort analysis. (c) Second cohort analysis.

Since only one miRNA reached statistical significance in the combined analysis, candidate miRNAs were selected from small RNA sequencing data according to a predefined prioritization strategy for subsequent RT-qPCR analysis (Table 2). hsa-miR-155–5p was designated as the primary candidate. Additional candidates were selected from the combined analysis based on statistical significance and fold change magnitude (effect size) (Fig. 2a), and from batch-specific analysis to ensure that relevant candidates were not overlooked (Figs. 2b–c). hsa-miR-375–3p was upregulated in both the second batch and the combined analysis, although in the latter it reached only nominal significance and did not pass multiple-testing correction. Finally, hsa-miR-512–3p, which did not meet the primary statistical threshold, was included due to prior evidence linking it to *T. cruzi* infection in placental explants (17) and its high expression level.

### Selection of reference miRNAs for RT-qPCR normalization

From the combined analysis, hsa-miR-455–3p (log_2_ fold change = 0.26; p-value = 0.148), and hsa-miR-99b-5p (log_2_ fold change = −0.09; p-value = 0.541) were selected as candidate reference miRNAs for subsequent validation of their suitability for RT-qPCR normalization. These were run together with the 10 selected candidates.

**Figure 3:**
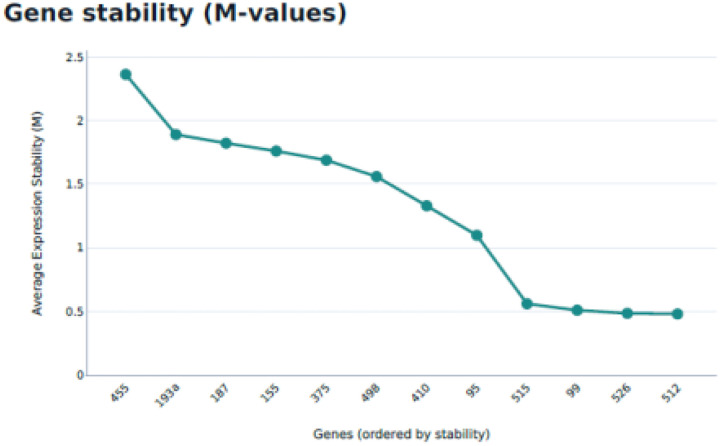
geNorm expression stability ranking of miRNAs evaluated for RT-qPCR normalization. The plot shows the average expression stability values (M) calculated using the geNorm algorithm for the 12 miRNAs evaluated by RT-qPCR. miRNAs are ordered from least stable to most stable according to their M values. Lower M values indicate greater expression stability. The three highest-ranked miRNAs were hsa-miR-512–3p, hsa-miR-526b-3p, and hsa-miR-99b-5p.

Expression stability was assessed across the 15 RT-qPCR samples using the geNorm algorithm. Pairwise variation analysis indicated an optimal normalization set of three reference miRNAs. The recommended set comprised hsa-miR-512–3p, hsa-miR-526b-3p, and hsa-miR-99b-5p, with a mean stability value (M) of 0.490 (Fig. 3 and Table S3), below the threshold of 0.5 for homogeneous sample panels according to Hellemans et al. (28). However, hsa-miR-526b-3p was excluded from the final normalization set because it showed a nominally significant between-group difference (p-value = 0.043). Therefore, RT-qPCR normalization was performed using hsa-miR-512–3p and hsa-miR-99b-5p.

The plot shows the average expression stability values (M) calculated using the geNorm algorithm for the 12 miRNAs evaluated by RT-qPCR. miRNAs are ordered from least stable to most stable according to their M values. Lower M values indicate greater expression stability. The three highest-ranked miRNAs were hsa-miR-512–3p, hsa-miR-526b-3p, and hsa-miR-99b-5p.

### RT-qPCR assessment of selected candidate miRNAs

**Figure 4:**
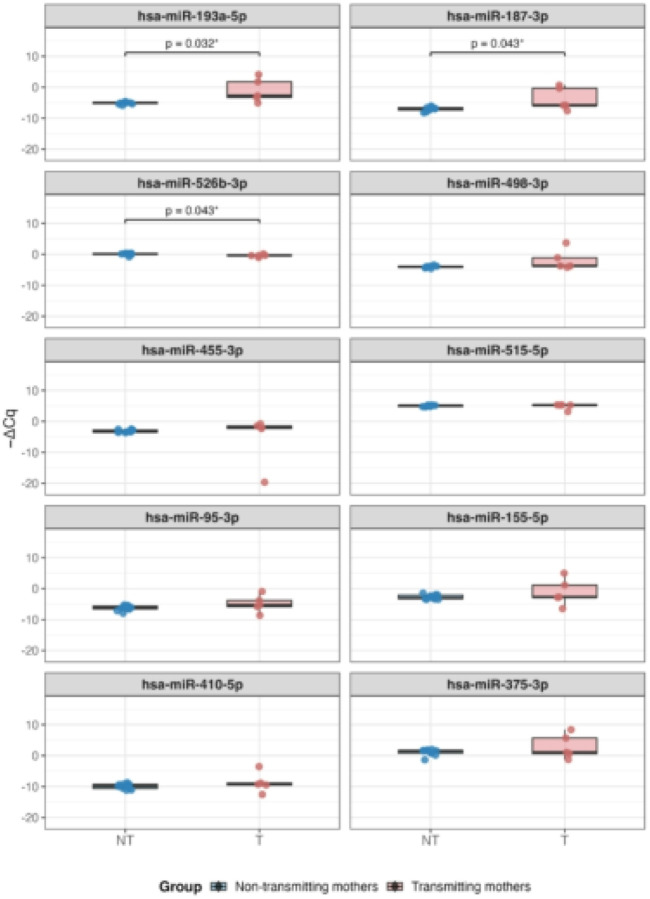
Distribution of - ΔCq values for selected candidate miRNAs assessed by RT-qPCR according to congenital transmission status. Boxplots show - ΔCq values for each candidate miRNA in placental samples from transmitting (T) and non-transmitting (NT) mothers. ΔCq values were calculated by normalizing the Cq value of each target miRNA to the mean Cq value of the selected endogenous reference miRNAs and then transformed to −ΔCq to facilitate visual interpretation. P-values from the Mann–Whitney U test are shown for the three miRNAs with p < 0.05; asterisks indicate p < 0.05.

RT-qPCR assessment of selected candidate miRNAs was performed in 15 placental samples, including 5 from transmitting mothers and 10 from non-transmitting mothers (Fig. 4 and Table 3). Among the evaluated candidates, hsa-miR-193a-5p and hsa-miR-187–3p showed significantly higher expression in placental samples from transmitting mothers compared with those from non-transmitting mothers, with log_2_ fold changes of 2.31 (4.95-fold; p = 0.032) and 1.53 (2.89-fold; p = 0.043), respectively.

ΔCq values are presented as mean ± standard deviation according to congenital transmission status. Fold change was calculated using the comparative Cq method as 2^-ΔΔCq, using the Hodges-Lehmann estimator to estimate the median of pairwise differences in ΔCq values between transmitting and non-transmitting mothers. Fold changes are presented with their corresponding 95% CIs. In the linear scale, fold change values > 1 indicate higher relative expression in transmitting mothers, whereas values < 1 indicate lower relative expression in transmitting mothers. Corresponding log_2_ fold change values are shown to facilitate comparison with the small RNA sequencing differential expression results.

hsa-miR-526b-3p also showed a significant between-group difference (p = 0.043), although the magnitude of the change in expression was small (log_2_ fold change = − 0.46; 0.73-fold). The remaining evaluated miRNAs did not show statistically significant differences in expression between groups (Fig. 4 and Table 3).

Boxplots show - ΔCq values for each candidate miRNA in placental samples from transmitting (T) and non-transmitting (NT) mothers. ΔCq values were calculated by normalizing the Cq value of each target miRNA to the mean Cq value of the selected endogenous reference miRNAs and then transformed to −ΔCq to facilitate visual interpretation. P-values from the Mann–Whitney U test are shown for the three miRNAs with p < 0.05; asterisks indicate p < 0.05.

**Figure 5:**
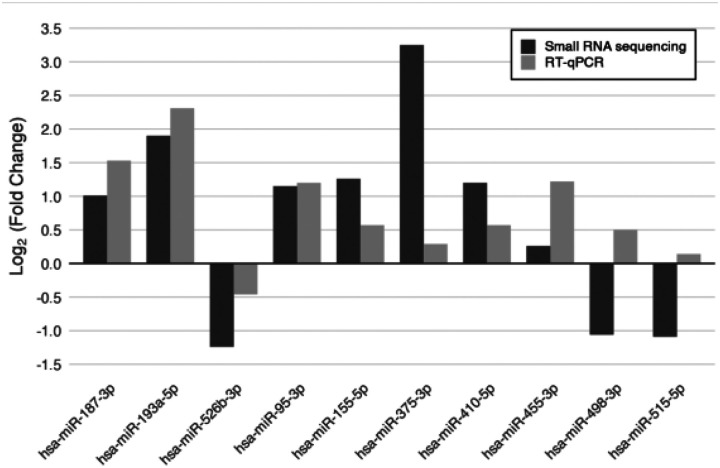
Comparison of estimated log_2_ fold changes obtained from small RNA sequencing and RT-qPCR for selected candidate miRNAs. Log_2_ fold change values on the y-axis greater than zero indicate upregulation in transmitting mothers relative to non-transmitting mothers, whereas values below zero indicate downregulation.

Overall, 8 of the 10 evaluated miRNAs showed concordant directionality between small RNA sequencing and RT-qPCR (hsa-miR-155–5p, hsa-miR-187–3p, hsa-miR-193a-5p, hsa-miR-375–3p, has-miR-410–5p, has-miR-455–3p, hsa-miR-526b-3p, and hsa-miR-95–3p). Of these, three candidates showed concordant directionality together with nominal statistical significance in both platforms (hsa-miR-187–3p, hsa-miR-193a-5p, and hsa-miR-526b-3p). Two miRNAs showed discordant directionality between platforms (hsa-miR-498–3p and hsa-miR-515–5p) (Fig. 5).

## DISCUSSION

This study explored placental miRNA expression profiles associated with congenital transmission of *T. cruzi* using small RNA sequencing, followed by RT-qPCR assessment of selected candidates in placentas from naturally infected mothers. By analyzing *in vivo*-derived human placental tissue, rather than experimental explants or *in vitro* models, this study provides crucial insights into host-parasite interactions at the maternal-fetal interface that controlled laboratory models often fail to capture. Our findings indicate that congenital transmission might not be associated with a global disruption of the placental miRNA expression profile, but rather with the dysregulation of a limited subset of miRNAs. The global analysis of placental miRNAs showed a broad repertoire shared between *T. cruzi*-transmitting and non-transmitting mothers (418 miRNAs), alongside smaller subsets of miRNAs detected uniquely in each group. This pattern is consistent with the notion of placental homeostatic resilience (31). Similar observations have been reported in other placental pathological conditions, such as preeclampsia and viral infections, where expression differences are concentrated in specific regulatory subsets rather than reflecting extensive miRNAome remodeling (14, 32).

In the small RNA sequencing analysis, hsa-miR-155–5p was the only miRNA that remained significantly upregulated in placentas from transmitting mothers after correction for multiple testing. As an immune-related miRNA involved in the regulation of inflammatory responses (33, 34), its increased expression in placental tissue under infection stress may reflect a finely tuned protective immune function and proinflammatory response at the maternal-fetal interface, aimed at controlling parasite replication. In support of this interpretation, miR-155 deficiency has been shown to enhance the susceptibility of C57BL/6 mice to *T. cruzi* infection by downregulating an effective Th1 response (35). In the present study, upon RT-qPCR analysis, hsa-miR-155–5p showed an expression fold change of 1.48. Although this difference did not reach statistical significance, its magnitude is not negligible and suggests potential biological relevance (36).

The RT-qPCR assessment provided complementary evidence for two additional candidates. hsa-miR-193a-5p showed the largest increase in relative expression in placental samples from transmitting mothers compared with non-transmitting mothers, with an estimated 4.95-fold difference (log_2_ fold change = 2.31). Members of the miR-193a family have been linked to apoptosis, cell-cycle regulation, and cellular stress responses, including regulation of MCL1 and Cyclin D1 (37, 38). These processes are relevant to placental tissue turnover, where trophoblast apoptosis and cellular stress have been implicated in placental dysfunction and host-pathogen interactions (39). hsa-miR-187–3p also exhibited higher relative expression in transmitting mothers, with an estimated 2.89-fold difference (log_2_ fold change = 1.53). This miRNA has been described as a negative regulator of inflammatory responses, including the modulation of NF-κB-related signaling and proinflammatory cytokine production(40) Such functions are consistent with a potential role in immune regulation at the maternal-fetal interface. Its upregulation in placental samples from *T. cruzi*-transmitting mothers may reflect a counter-regulatory response or a parasite-driven immune evasion mechanism. Regardless, the specific roles of these miRNAs in placental *T. cruzi* infection remain to be established. The results presented here support their consideration as candidates for further evaluation rather than as confirmed biomarkers or direct contributors to congenital transmission.

Some of the selected candidates belong to the placenta-enriched C19MC cluster, including hsa-miR-512–3p, hsa-miR-515–5p, and hsa-miR-526b-3p. The C19MC cluster, located on chromosome 19, is the largest placenta-enriched miRNA cluster in humans and is predominantly expressed in trophoblast cells. It has been implicated in placental development, cell differentiation, proliferation, and intercellular communication through extracellular vesicles (31, 41), including the transfer of trophoblast-derived exosomes that can induce antiviral resistance in recipient cells (31). Previous *ex vivo* studies using human placental explants infected with *T. cruzi* have reported modulation of C19MC-related miRNAs involved in trophoblast differentiation, apoptosis, inflammatory responses, and NF-κB signaling (16, 17) Guerrero-Muñoz et al. (42) identified miR-512–3p as involved in *T. cruzi*-induced apoptosis through regulation of c-FLIP, a caspase-8 inhibitor. In contrast, the present study did not detect significant changes in expression (log_2_ fold change < 1) for any of the C19MC candidates assessed by RT-qPCR when comparing *T. cruzi*-transmitting and non-transmitting placentas. This discrepancy may reflect differences in biological context between acute, controlled *ex vivo* models and naturally infected placentas from chronically infected patients. In the latter, long-term host-parasite interactions, heterogeneity in placental tissue composition, and physiological changes at the maternal-fetal interface may attenuate or mask localized, cell type-specific C19MC-related signals. In line with this interpretation, recent placental bulk RNA-seq data from naturally infected pregnancies have shown that congenital *T. cruzi* transmission is associated with broad transcriptional changes involving trophoblast-related signatures, cell adhesion, extracellular matrix organization, and placental barrier integrity rather than isolated molecular alterations (43). Although that study did not directly assess mature miRNAs, it supports the notion that the *in vivo* placental response is shaped by complex tissue-level and cell-type-dependent processes (43). Taken together, these findings underscore that *ex vivo* models and *in vivo*-derived placental studies provide complementary insights into placental responses to *T. cruzi* infection.

From an analytical perspective, the use of DESeq2 (44) with FDR correction and adjustment for sequencing batch effects, strengthens confidence in the reported findings by reducing false-positive discoveries across the hundreds of miRNAs tested simultaneously. Discrepances between the two platforms used for quantification of miRNA expression reflect, in part, fundamental methodological differences: small RNA sequencing estimates differential abundance based on globally normalized read counts across the detected miRNA repertoire, whereas RT-qPCR measures relative transcript abundance of selected target genes between samples through normalization to stably expressed endogenous reference genes. The latter provides superior sensitivity and accuracy for the quantification of miRNAs, even at low copy numbers (45). Importantly, 8 of the 10 evaluated miRNAs in the present study showed concordant expression trends between small RNA sequencing and RT-qPCR, with three reaching statistical significance in the latter.

This study has limitations that should be considered when interpreting the findings. First, the sample size was limited for both analyses, particularly for RT-qPCR, which may have reduced the statistical power to detect biologically meaningful differences. This limitation reflects the inherent challenges of collecting placental samples from well-characterized cohorts of infected mothers with documented congenital transmission outcomes. Second, placental samples were obtained from two cohorts recruited during different study periods and processed in separate sequencing batches. Although both cohorts were recruited at the same hospital using comparable study designs, and both transmitting and non-transmitting samples were represented in each batch, transmission status was unevenly distributed across batches. Although batch was included as a covariate in the DESeq2 model, concordance between batch-specific results was limited. Residual batch- or cohort-related effects on the differential expression findings cannot be excluded and should be considered when interpreting the sequencing results. Finally, the RT-qPCR assessment was not performed on a fully independent sample set, as it included samples that overlapped with the sequencing set, together with two additional samples not included in the sequencing analysis. Therefore, the RT-qPCR analysis should be interpreted as an exploratory assessment of selected candidates rather than as an independent validation.

A notable strength is the use of placental tissue obtained at delivery from naturally infected mothers. Although placentas are generally discarded after birth, samples from women with confirmed *T. cruzi* infection during pregnancy are difficult to obtain, making this biological material both valuable and clinically meaningful. This is particularly relevant given that most available evidence on placental miRNA responses to *T. cruzi* derives from *ex vivo* infection models. The fact that the present study analyzed tissue from naturally infected pregnancies directly strengthens the translational potential of the findings. A further methodological strength is that transmitting and non-transmitting groups were matched by maternal age and mode of delivery, variables previously associated with congenital *T. cruzi* transmission (46), thereby reducing the likelihood that these factors confounded the observed differences in placental miRNA expression.

## CONCLUSIONS

In conclusion, placental miRNA expression profiling of naturally infected pregnancies identified hsa-miR-155–5p, hsa-miR-193a-5p, and hsa-miR-187–3p as candidate miRNAs associated with congenital *T. cruzi* transmission. These showed consistent directionality across small RNA sequencing and RT-qPCR data despite incomplete statistical concordance between platforms. Together, these findings suggest that congenital transmission may be associated with changes in the expression of selected placental miRNAs rather than with a broad disruption of the global miRNA profile, a distinction that could be relevant for understanding the placental biology during *T. cruzi* transmission. Larger studies using well-characterized placental cohorts, transcriptomic approaches, and functional analyses are needed to validate these candidates, clarify their regulatory roles, and evaluate their potential as biomarkers of congenital transmission.

## Supplementary Material

Supplementary Files

This is a list of supplementary files associated with this preprint. Click to download.

- AdditionalmaterialTablesS1S3.docx

## Figures and Tables

**Table 1:** Maternal and neonatal characteristics of the study population according to congenital transmission status

Characteristics	Transmitting (n = 13)	Non-transmitting (n = 18)	Total (n = 31)	p-value
Maternal age, years⊠	31 (27–35)	29 (27–35)	30 (27–35)	0.56⊠
Number of pregnancies⊠	4 (3–4)	4 (3–5)	4 (3–4)	0.61⊠
Number of live births⊠	4 (3–4)	4 (3–4)	4 (3–4)	0.67⊠
Mode of delivery⊠				1.00⊠
Vaginal delivery	1 (7.69)	2 (11.11)	3 (9.68)	
Cesarean delivery	12 (92.31)	16 (88.89)	28 (90.32)	
Newborn sex⊠				0.13⊠
Male	10 (76.92)	9 (50.00)	19 (61.29)	
Female	3 (23.08)	9 (50.00)	12 (38.71)	
Neonatal peripheral blood qPCR result⊠				
Positive	9 (69.23)	0 (0.00)	9 (29.03)	
Negative	0 (0.00)	18 (100.00)	18 (58.06)	
Not performed	4⊠ (30.77)	0 (0.00)	4 (12.90)	
Neonatal cord blood qPCR result⊠				
Positive	8 (61.54)	0 (0.00)	8 (25.81)	
Negative	1⊠ (7.69)	18 (100.00)	19 (61.29)	
Not performed	4⊠ (30.77)	0 (0.00)	4 (12.90)	

⊠ Data are presented as median (interquartile range).

⊠ Data are presented as n (%).

⊠ Newborns were positive by qPCR in cord blood samples.

⊠ Newborns were positive by qPCR in peripheral blood samples.

⊠ Wilcoxon rank-sum test.

⊠ Fisher’s exact test.

⊠ Chi-square test.

**Table 2:** Candidate miRNAs selected from small RNA sequencing for RT-qPCR assessment

Source analysis	Candidate miRNA	baseMean (reads)	Direction in transmitting placentas	Log_2_ fold change	Statistical evidence	Selection criteria
Combined analysis	hsa-miR-155–5p	1627.8	Upregulated	1.26	0.024^a^	Adjusted p-value, effect size, expression abundance
Combined analysis	hsa-miR-498–3p	38.9	Downregulated	−1.06	0.003^b^	Nominal p-value, effect size
Batch 1	hsa-miR-515–5p^c^	6968.1	Downregulated	−1.09	0.001^b^	Nominal p-value, effect size, expression abundance
Batch 1	hsa-miR-526b-3p	63.4	Downregulated	−1.24	0.003^b^	Nominal p-value, effect size
Batch 1	hsa-miR-95–3p	284.2	Upregulated	1.15	0.004^b^	Nominal p-value, effect size
Batch 1	hsa-miR-410–5p	14.7	Upregulated	1.20	0.013^b^	Nominal p-value, effect size
Batch 1	hsa-miR-187–3p	17.2	Upregulated	1.01	0.021^b^	Nominal p-value, effect size
Batch 2	hsa-miR-375–3p^d^	284.1	Upregulated	3.25	< 0.001^a^	Adjusted p-value, effect size
Batch 2	hsa-miR-193a-5p	5529.7	Upregulated	1.90	< 0.001^a^	Adjusted p-value, effect size, expression abundance
	hsa-miR-512–3p	1297.2	Downregulated	−0.34	0.126^b^	Previous evidence in *T. cruzi*-infected placental explants(17), and high expression abundance

a Adjusted p-value,

b Nominal p-value,

c has-515–5p emerged in Batch 2 with a baseMean = 2230.45, log_2_ fold change = 1.173, and nominal p-value = 0.014.

d hsa-miR-375–3p emerged in the combined analysis with a baseMean = 165, log_2_ fold change = 1.529, nominal p-value = 0.001, and adjusted p-value = 0.166. Abbreviations: baseMean, mean normalized read count across samples (indicator of miRNA expression abundance); log_2_ fold change, base 2 logarithm of the fold change; miRNA, microRNA; RT-qPCR, reverse transcription quantitative polymerase chain reaction.

**Table 3:** ΔCq values, fold change, log_2_ fold change, and p-value of selected candidate miRNAs assessed by RT-qPCR

miRNA	Mean ΔCq ± SD, non-transmitting group	Mean ΔCq ± SD, transmitting group	Fold change, HL estimator (95% CI)	Log_2_ fold change	p-value⊠
hsa-miR-193a-5p	5.11 ± 0.36	1.06 ± 3.83	4.95 (1.26–482.73)	2.31	0.032
hsa-miR-187–3p	7.05 ± 0.69	3.80 ± 3.71	2.89 (1.13–159.79)	1.53	0.043
hsa-miR-526b-3p	−0.07 ± 0.34	0.38 ± 0.41	0.73 (0.51–0.98)	−0.46	0.043
hsa-miR-498–3p	4.05 ± 0.32	1.81 ± 3.31	1.42 (1.00–183.55)	0.50	0.058
hsa-miR-455–3p	3.11 ± 0.44	5.22 ± 8.07	2.34 (1.37×10^−5^–4.06)	1.22	0.076
hsa-miR-515–5p	−5.04 ± 0.21	−4.88 ± 0.95	1.10 (0.33–1.40)	0.14	0.198
hsa-miR-95–3p	6.28 ± 0.82	4.92 ± 2.82	2.29 (0.35–22.39)	1.20	0.198
hsa-miR-155–5p	2.75 ± 0.75	1.19 ± 4.38	1.48 (0.12–122.37)	0.57	0.426
hsa-miR-410–5p	9.91 ± 0.85	8.72 ± 3.25	1.49 (0.35–50.92)	0.57	0.426
hsa-miR-375–3p	−1.10 ± 1.03	−2.93 ± 3.99	1.22 (0.37–103.61)	0.29	0.854

⊠ p-values were obtained using the Mann–Whitney U test.

HL, Hodges–Lehmann; CI, confidence interval.

## Data Availability

The small RNA sequencing dataset was deposited in the Gene Expression Omnibus (GEO) database (https://www.ncbi.nlm.nih.gov/gds) under the accession number GSE333874.Data from RT-qPCR and associated variables will be available upon request.

## Abbreviations

miRNA: MicroRNA
cDNA: Complementary DNA
PCA: Principal component analysis
ANOVA: Analysis of variance
FDR: False discovery rate
RINe: RNA integrity number equivalent
C19MC: Chromosome 19 microRNA cluster
Cq: Quantification cycle
qPCR: Quantitative polymerase chain reaction
RT-qPCR: Reverse transcription quantitative polymerase chain reaction
ELISA: Enzyme-linked immunosorbent assay
IQR: Interquartile range
NF-kB: Nuclear factor kappa B
